# Centroid analysis: Inferring concept representations from open-ended word responses

**DOI:** 10.3758/s13428-026-03031-y

**Published:** 2026-04-29

**Authors:** Aliona Petrenco, Fritz Günther

**Affiliations:** Department of Psychology, Humboldt-Universität zu Berlin, Unter den Linden 6, Berlin, 10099 Germany

**Keywords:** Distributional semantics, Mental representations, Concepts, Centroid analysis

## Abstract

The present research proposes and evaluates a novel method – centroid analysis – for measuring representations and concepts at both individual and group levels by mapping open-ended responses onto a pre-existing semantic vector space. Centroid analysis allows us to retrace the target concept as the geometric center of the semantic vectors of the responses generated by this concept. At the group level, centroid analysis enables researchers to compare conceptual structures across different populations to investigate how factors such as language, culture, cognitive differences, educational background, or exposure to specific narratives shape shared representations. At the individual level, centroid analysis enables fine-grained assessments of how personal experiences, expertise, cognitive styles, and even temporary contextual influences affect conceptual representations. We evaluate this method using two distributional semantic models across several calculation methods, reference lexicon sizes, response types, and datasets with tasks ranging from single-word substitutions to single and multiple free associations and multiple feature generation. We conclude that at the group level, the best method to retrace the response-generating concept as a vector in a multi-dimensional semantic space from the averaged vectors of participant responses is to collect multiple free associations (70 unique and 245 total responses per target), use fastText for meaning-to-vector mapping for responses and targets, and to consider each response in the centroid calculation as often as it occurred in the data. At the individual level, the best results are achieved by employing fastText and considering at least eight responses per item per participant in the centroid calculation.

Measuring and quantifying theoretical constructs is a central endeavor in all empirical sciences. It enables researchers to formulate and test precise hypotheses that allow conclusions about theoretical assumptions. In psychology, such measurements are especially difficult because virtually all relevant theoretical constructs are not directly observable. Instead, we need to infer them from other phenomena we assume are affected by these constructs, such as behavioral responses to specific tasks. This is the core objective of the field of psychometrics, where for example the intensity of a sensation is estimated from choices between different response options (such as “which stimulus is more intense on a given dimension”; Fechner, 1860; Stevens, 1975), or underlying abilities are estimated from a behavioral performance on tasks related to that ability (typically, whether the task is solved or not; Rasch, 1980).

One of the central theoretical constructs in cognitive psychology is mental representations (Quilty-Dunn et al., 2023), which Smith , (1998, p. 391) defines as “an encoding of some information, which an individual can construct, retain in memory, access, and use in various ways“. Mental representations of categories (which are themselves sets of entities) are referred to as *concepts*, which constitute the building blocks of semantic memory and organize our knowledge about the world and the things in it Murphy (2002); Kumar (2021); Yee et al. (2018).

It does not make much sense to measure and compare mental representations and concepts on a single scale. Instead, more sensible measures for comparison are either the value of a representation on a given semantic dimension, such as, for example, valence (Bradley & Lang, 1999; Osgood et al., 1957; Warriner et al., 2013), or the overall similarity between representations (Goldstone & Son, 2012; Roads & Love, 2021, 2024; Wingfield & Connell, 2023). Both types of comparison are realized in a vector space format, where each mental representation or concept is represented by a (usually high-dimensional) numerical vector of semantic dimensions (Bradley & Lang, 1999; Cassani et al., 2024; Günther et al., 2023; Hebart et al., 2020; Landauer & Dumais, 1997; Lynott et al., 2020; Osgood et al., 1957; Piantadosi et al., 2024; Roads & Love, 2024). Implementations of such semantic vector space models come in at least two varieties: Some are constructed directly from behavioral data, such as participant ratings on given meaning dimensions (Bradley & Lang, 1999; Lynott et al., 2020; Osgood et al., 1957) or similarity judgments (Hebart et al., 2020; Roads & Love, 2021), while others are constructed in a data-driven manner, by applying some learning algorithm to a proxy of experience (Günther et al., 2023; Landauer & Dumais, 1997; Lenci et al., 2022; Mandera et al., 2017).

Typically, these vector space models present a general, popu-lation-level representation system (Günther et al., 2019). However, general-level representations are not suited to investigate research questions that require measuring concepts in a specific (group of) individual(s) and in a specific situation and context. For example, one may imagine a survey in which researchers want to compare the representations for concepts such as *freedom*, *justice*, or *responsibility* between participants who identify as left-wing or right-wing, or who have just read left-wing or right-wing propaganda material. Alternatively, one may want to measure the effect of different teaching materials or classroom instructions on students’ representations of concepts such as *life* or *science*. Such examinations do not need to be restricted to lexicalized concepts: One may be interested in how different groups represent different historical events based on their age or social group identity, or how different individuals represent what is expressed in an ambiguous piece of modern art, depending on whether they were provided with context information about the artist and the historical context. Furthermore, one might be interested in how the same individual’s conceptual representations shift when communicating across distinct discourse domains.

Indeed, research into the relativity of word meaning demonstrates measurable conceptual variation in the meanings of common words across different languages and within the same language among different individuals. For instance, Thompson et al. (2020) uses large-scale cross-linguistic semantic neighborhood analysis of 1010 distinct concepts in 41 languages and finds conceptual variation in languages with low geographical, historical, and cultural proximity. On a within-language scale, Johns (2024) examines corpora comprising the text production of 500 individual Reddit users and observes, among others, differences in word meanings in distinct individuals but also the same individuals communicating across distinct discourse topics. Similarly, Marti et al. (2023) uses similarity and feature judgments to demonstrate that conceptual spaces differ across individuals and that such variability exists even when the same label is used to denote different concepts. Finally, using Semantic distance-judgments and neuroimaging data, Wang and Bi (2021) find intersubject shifts in conceptual representations as a function of sensory-motor and descriptive attributes of the conceptual referent.

From a theoretical standpoint, these findings reveal variability in individual and group meaning representations, which is shaped by differences experiential, linguistic, cultural, historical, and geographical environments, alongside language users’ individual communication patterns. From a practical standpoint, these findings emphasize the importance of developing methodological approaches suitable for investigating the variability of experiential concept meaning and the contextual fluidity. At the same time, addressing such variability requires less data-intensive methods, as large-scale corpora are often unavailable for individual speakers or specific contexts.

Building on these considerations, in the present article we propose and evaluate a scalable method for investigating word meaning variability as a function of culture, social and communicative environments, and group or individual identity. Specifically, this method allows the measurement of representations and concepts in a specific (group of) individual(s) and in a specific situation and context by mapping them into a pre-existing semantic vector space. This is achieved by collecting and analyzing open-format verbal responses to the concept of interest. More specifically, we demonstrate that the concept evoking verbal responses in an open-response format, such as a free-association task, can be inferred as the centroid (the geometric center) of the semantic vector representations of those responses. As a shortcut, we refer to this method as *centroid analysis*. Importantly, the result of this computation will be placed in a general-level semantic space and can thus be compared to all concepts available in that space, as well as to all other concepts measured via centroid analysis. As outlined in the *Practical considerations* section below, this method is especially useful for comparing specific concept representations to all other concepts in that general-level semantic space while remaining an economical method to assess individual-level or situation-specific representations.

## Theoretical underpinnings

On a theoretical level, centroid analysis relies on the assumptions that (a) language production starts in a setting involving a prior presentation of a linguistic stimulus, (b) language production entails the activation of the target concept in semantic space, and (c) that the responses are then activated via a spreading-activation mechanism originating from that target concept (Batali, 1998; Pugacheva & Günther, 2024). Suppose that is the case and activation spreads evenly in all directions of the semantic space. The representation or concept at the origin of that activation should then be retrievable as the center of all responses – similar to determining the center of an earthquake as the geographic center of the sites that have been affected. Evidence for this assumption has recently been provided by Pugacheva and Günther (2024):

In their taboo-game paradigm, Pugacheva and Günther (2024) presented participants with single target words (such as *dragon*), and instructed them to convey the meaning of those words so that another person could understand them, but without using the target words (with responses such as *beast* or *wyvern*). The task was thus to produce single-word substitutions that could subsequently be identified by recipients. Participants were instructed that novel word responses (such as *firelizard*) were also permitted. In their analysis, the authors first computed semantic similarities between target words and each individual participant’s response as the cosine similarities of their distributional vector representations (Bojanowski et al., 2017; Marelli et al., 2017; Mikolov et al., 2013). To allow the comparison across semantic spaces of different dimensionality, the authors derived semantic neighborhood ranks for each response from these cosines. For instance, if a response showed the highest cosine similarity to the target word (compared to the 20,000 most frequent words in the given space), it received a rank of 1; if 100 other words had a higher cosine similarity, it received a rank of 101. The overwhelming majority of responses received very low ranks and were thus very close to the respective target word.

In a next step, to examine whether a given target can be uniquely identified from the entire set of responses it produced, Pugacheva and Günther (2024) presented the *centroid analysis* technique. The authors pooled all response vectors for a given target word, computed their centroid (i.e., the geometric center or average vector), and calculated the rank of each target word in the neighborhood of this centroid. The target was considered retrieved if it was the closest word to its corresponding response centroid (i.e., it had the neighborhood rank of 1). In their Experiment 1 (in which participants were free to produce both novel and existing words as responses, e.g., *firelizard* and *beast* correspondingly), the target word was the closest word to the response centroid in Hit@1 = 38.6%^1^ of cases (Hit@5 = 73.4% within the first five, Hit@20 = 91.1% within the first twenty neighbors – which still the top 0.1% of words in the lexicon). These findings indicate that, in many cases, the intended meaning (the concept evoking a specific set of responses) can be well captured and recovered from the centroid of participants’ responses. This suggests that centroid analysis is a suitable method for investigating concept meaning representations in groups of speakers.

Here, we follow up on these initial analyses in two key directions. On the one hand, we conduct a systematic evaluation of the centroid analysis method across multiple task settings and with different hyper-parameters, with the goal of identifying the experimental task settings and analytical parameter combinations that most accurately recover meaning of a concept from a set of verbal responses. On the other hand, we extend the scope of this evaluation beyond the group-level, collecting and analyzing data that enable the investigation of meaning representations between and within individual speakers.

## Practical considerations

Centroid analysis is especially useful and relevant when working with large-scale data-driven models: For example, distributional semantic models/word embedding models/language models (Lenci et al., 2022) – which currently take a highly prominent position as computational models of semantic memory (Günther et al., 2019; Kumar, 2021; Yee et al., 2018) and perform particularly well in predicting different types of behavior (Hussain et al., 2024) – are constructed by learning distributional patterns of words in large collections of natural text. They thus provide general, population-level vector representations of concepts, but clearly lack an in-built method to measure group- or individual-level concepts in specific contexts or situations: One will not have access to the text data representing the language experience (or output) of a given participant in an experimental study; and even if this were the case, this would still not allow to measure the effects of a specific experimental manipulation, as described in the different examples above. This is the crucial methodological gap that centroid analysis addresses and closes.

On the other hand, open responses such as free associations are relatively easy and fast to collect, which makes the method of collecting responses, mapping them into a semantic space, and calculating their centroid convenient and affordable. In addition, the open verbal response format comes very natural to most participants, also when compared to other methods such as numerical ratings on multiple semantic dimensions that can sometimes appear arbitrary and counter-intuitive (Kjell et al., 2019). Thus, while some behavior-based vector space models inherently enable the measurement of individual-level and situation-dependent concept representations (for example, collecting ratings for “freedom” on a semantic differential from participants who identify as left-wing versus right-wing), a measurement via open responses may be more valid and reliable in these cases (Kjell et al., 2019).

## Centroid analysis: Setup for a systematic evaluation

### The algorithm

The algorithm for a centroid analysis is simple and straightforward, and consists of the following steps:

1. Collect open-format verbal responses to the target concept in question. This can be done with different instructions, such as free association (De Deyne et al., 2019; Nelson et al., 2004), word substitution (Pugacheva & Günther, 2024), or feature generation (Buchanan et al., 2013, 2019).
2. Collect semantic vectors/word embeddings for the individual responses (for more details on different models, see *Hyperparameters* below).
3. Compute the centroid (= average vector) of these individual response vectors as the estimate for the target concept representation.

### Hyperparameters

There are two hyperparameters in the algorithm described above, which the present study will systematically explore:

#### The semantic space

In principle, a centroid analysis can be performed with *any* model that represents word meanings or concepts as numerical vectors or *word embeddings* (e.g., Fritz Günther et al., 2023; Lenci et al., 2022). The most prominent class of such models is *distributional semantic models (DSMs)* or *word embedding models* (Günther et al., 2019; Jones et al., 2015; Kumar, 2021; Landauer & Dumais, 1997). Based on the distributional hypothesis that words with similar meanings appear in similar contexts (Firth, 1957; Harris, 1954), these models approximate word meanings via their distributional patterns over contexts (such as other words or documents) in large text corpora (Lenci, 2008; Sahlgren, 2008). In the present research, we employ two models that have shown good empirical performance in previous studies (Baroni et al., 2014; Mandera et al., 2017; Pugacheva & Günther, 2024): The *word2vec* model (Mikolov et al., 2013) and the *fastText* model (Bojanowski et al., 2017). Both are prediction-based models that are trained to predict a word from the other words immediately surrounding it, using a neural network architecture with one hidden layer. After model training is completed, the activation values in the hidden layer when any given word in the input layer is activated (which is equivalent to the weights between that input word and the hidden layer) are then taken as the word embedding (i.e., vector representation) of that word’s meaning. More specifically, we employ:

- The *word2vec* model with the best empirical performance in a large-scale evaluation study by Baroni et al. (2014), trained using the *cbow* algorithm with 400-dimensional embeddings, negative sampling with $k = 10$, and subsampling with $t = 1e^{-5}$. This model was trained on a $\sim$ 2 billion word concatenation of the ukWaC web corpus (Baroni et al., 2009), a Wikipedia dump, and the British National Corpus (BNC Consortium, 2007), and contains word embeddings for 300,000 different words.
- A *fastText* model with subword information trained on the 600-billion-word Common Crawl corpus, downloaded from https://fasttext.cc/docs/en/english-vectors.html (Mikolov et al., 2018). This model originally contains 2 million different 300-dimensional word embeddings. For our purposes, we extracted embeddings for all 300,000 words that also appear in the word2vec model by Baroni et al. (2014).

#### Handling non-unique responses

Especially when open responses are collected from different participants, at least two participants likely produce the same response. When computing the centroid, we have to decide whether to perform the computation at the token level (including each response as many times as it was produced) or at the type level (including each unique response only once). The first option realizes the rationale that a word that is closer to a target will be produced more often and should therefore be weighted more; the second option weighs each unique response equally, irrespective of how often it was produced, and thus more strongly emphasizes the information provided by rare responses.

## Evaluation

### Datasets

We propose centroid analysis as a method for estimating concept representations from which participants produce open responses. To systematically and quantitatively evaluate this method, we use existing data from studies that elicit participants’ responses to a given target word. This allows us to take the target word as a gold standard that has to be recovered/correctly identified from the responses via centroid analysis.

Specifically, we use the following datasets for evaluation:

#### Group-level

 

- The first dataset, henceforth ***Taboo***, includes target–response pairs collected in a taboo game paradigm, where participants are presented with a target word and have to express the meaning of this word with a single other word. This dataset pools data from three separate experiments employing the same experimental paradigm: In Pugacheva and Günther (2024, Experiment 1) and Raveling and Günther (2026), participants were free to also produce novel words as responses; in Pugacheva and Günther (2024, Experiment 2), participants were explicitly instructed in each trial to either produce an existing or a novel word. The pooled dataset from all three experiments includes 28,317 responses (17,368 existing words and 10,949 novel words) to 1463 different target words, with 20 to 51 responses ($M = 34.8$) and 2 to 46 unique responses ($M = 24.2$)^2^ per target. The data by Pugacheva and Günther (2024) were collected from 394 participants (200 in Experiment 1, 194 in Experiment 2), each of whom was presented with 48 out of 383 different target words. The data by Raveling and Günther (2026) – a replication of Pugacheva and Günther (2024, Experiment 1) with different target words – were collected from 480 participants, each of whom was presented with 54 out of 1080 different target words. Each participant produced *one response* per word.
- The University of South Florida Free Association norms (Nelson et al., 2004), henceforth ***Nelson***, are a seminal resource in research on free associations. Originally, this dataset contains 612,752 responses to 5019 different target words, with 15 to 185 responses ($M = 122.1$) and 1 to 34 unique responses ($M = 14.4$) per target. After the exclusion of targets and responses that were not mapped in our semantic models (specifically, the word2vec model, since vectors for all character strings can be extracted from fastText), the dataset used in our evaluation includes 602,832 responses to 4080 different target words, with 15 to 185 responses ($M = 122.0$) and 1 to 34 unique responses ($M = 14.4$) per target. According to Nelson et al. (2004), data were collected from more than 6000 participants. Booklets containing 100–120 words were given to $M = 149$ $(\textit{SD} = 15)$ participants each. Importantly, each participant was asked to provide *only a single response* per word.
- The Small World of Words dataset (De Deyne et al., 2019), henceforth ***SWoW***, is a large-scale dataset of free associations. Originally, this dataset^3^ contains 2,401,336 responses to 8661 different target words, with 64 to 300 responses ($M = 277.3$) and 35 to 192 unique responses ($M = 113$) per target. After excluding targets and responses that were not mapped in the word2vec model, the dataset used in our evaluation includes 2,075,318 responses to 8091 target words, with 56 to 297 responses ($M = 256.5$) and 32 to 175 unique responses ($M = 98.6$) per target. De Deyne et al. (2019) describes that they collected data from 88,722 participants, each of whom was presented with 14 to 18 different target words. Importantly, in contrast to Nelson et al. (2004), participants were instructed to produce *up to three responses per target* (but had the option to proceed without producing all three). Therefore, this dataset is expected to contain more responses that are less strongly associated with their target words.
- The English semantic feature production norms (Buchanan et al., 2019), henceforth ***Buchanan***, contains data collected with the specific instruction to produce features of the presented concepts. The resulting responses could consist of multiple words (such as *has feathers*) and have different inflectional forms (e.g., *feather*, *feathers*). After data pre-processing, Buchanan et al. (2019) provides a dataset where each produced feature is expressed as a single root word (in a column labeled *translated*). The original final dataset contains 498,321 responses to 4436 different target words, with 18 to 1977 responses ($M = 172.3$) and 5 to 62 unique responses ($M = 17.7$) per target. After the exclusion of targets and responses that were not mapped in the word2vec model, the dataset used in our evaluation includes 493,049 responses to 4396 different target words, with 18 to 1951 responses ($M = 172.5$) and 4 to 62 unique responses ($M = 17.7$) per target. Buchanan et al. (2019) expanded the feature norms originally collected by Buchanan et al. (2013), roughly doubling the number of targets. Across both studies, data were collected from 3065 participants; the number of targets presented to each participant varied across different data collections (for more details, see Buchanan et al., 2019, Table 1). Participants were instructed to generate features for each concept and were provided with examples of features and examples for possible responses to *duck*. Participants were asked to generate *as many features as they could think of* but to produce at least a few.

#### Individual-level

 

- To examine centroid analysis as a method to measure individual-level concept representations derived from a single individual’s responses, we collected our own free association data. Using the online platform Prolific (https://www.prolific.com/), data were collected from 281 participants who were instructed to produce ten associations for each of the 15 target words they were presented with. In total, we collected 42,150 participant responses to 210 target words with *ten responses* per target word per participant. All responses were run through the spell checker R library Hunspell, using British and second US English spelling conventions. The final dataset, henceforth ***individual-level***, includes 42,015 responses with 8–10 unique responses per target per participant ($M= 9.9$). The description of the experiment and the instructions were adapted from De Deyne et al. (2019).

### Evaluation setup

To evaluate the centroid analysis, we extracted all responses for a given target word in a given dataset and calculated the centroid of the word embeddings for these responses in a given semantic model (fastText or word2vec). For instance, in the taboo dataset, the response centroid for the target word *relativity* was obtained by averaging the word embeddings for the collected word substitutions like *spacecongruence*, *similarity*, and *relevance* (single response/participant). In the Buchanan dataset, the centroid for *relativity* was calculated as an average of embeddings for the produced features like *close*, *compare*, and *relate* (multiple responses/participant). In the SWoW and Nelson datasets, the meaning representation of the concept *relativity* was approximated by averaging the embeddings of free association responses like *space*, *time*, and *gravity*. Importantly, while SWoW allowed multiple responses (up to three) per target, Nelson collected single responses. Similarly to SWoW and Nelson, in the individual dataset, the embeddings of the free associations like *nephew*, *cousin*, and *time* were averaged to approximate the representation of the target concept in the semantic space. However, since in the latter case we were interested in individual-specific concept representations, centroids for each item were computed from each participant’s set of up to ten unique responses separately, rather than averaged across participants as in the group-level datasets. In all four group-level datasets, non-unique responses were considered in the centroid calculation either once or as often as they were generated.

After computing the response centroids, we calculated $\cos {(\text {centroid, target})}$, the cosine similarity between the resulting centroid and the target word, which is taken as the gold standard to be recovered by the centroid. We note here that this approach relies on simplified assumptions, namely (1) that these type-level population-level word embeddings for targets and responses are sufficiently reliable approximations of the respective underlying semantic representations of groups or individual speakers, and (2) that these representations are the same between different groups or speakers. Under these assumptions, cosine similarity provides a natural first benchmark for evaluating how well the centroid recovers the target representation.

In an ideal case, the cosine similarity between the centroid of responses and the word embedding for the target would always be equal to 1, indicating ideal vector overlap, but empirically, this is extremely unlikely to happen. At this point, relying only on cosine similarity is not ideal: Even though cosine simila-rity is technically a standardized measure that is bounded between 0 (completely unrelated) and 1 (identical), in reality, the absolute level of cosine similarity can differ substantially between different semantic spaces, and depending on the semantic space, even the cosine similarities to the nearest possible meanings are not necessarily extremely high: for example, in our fastText space, we find $$\cos {(\text {lion, lions}) = .781}$$, $$\cos {(\text {pa-}}$$$$\text {racetamol, ibuprofen}) = .794$$, or $$\cos \,(\text {Berlin, Germany}) = .719$$.

To arrive at a more meaningful and comparable metric of how well the response centroid captures the intended meaning of the target concept, instead, we used cosine similarities to determine *Hit@k* values via neighborhood ranks (see also Pugacheva & Günther, 2024). Specifically, after computing the cosine similarities between the response centroid and the target word, we calculated the cosine similarities between the response centroid and the $N = 20,000$ (50,000/100,000/250,000) most frequent English words (according to the corpus used by Baroni et al., 2014). The number of most frequent English words, or the lexicon size, allows us to examine the robustness of our results, as the increase in the reference lexicon size inevitably leads to a decrease in Hit@k values. We ranked all the cosines from highest to lowest to determine the target’s position within the centroid’s semantic neighborhood, with lower ranks indicating greater proximity between the two. For example, a neighborhood rank of 3 for the target word indicates that only 2 other existing words are closer to the response centroid than the target.

Next, we used the resulting neighborhood ranks to compute Hit@k values, reflecting how often the target (e.g., *relativity*) appeared among the top-k nearest neighbors (k = 1–20) of its response centroid. Higher Hit@k values indicate that targets more frequently occupy top positions in their centroid neighborhoods, corresponding to stronger alignment between the semantic representation of the centroid and target. Within this context, if the centroid allows us to correctly identify the target from which it was produced, then $\cos {(\text {centroid, target})}$ should be higher than all the other cosine similarities with which it was contrasted. In other words, ideally, the rank of the target in the neighborhood of the response centroid should always be 1, corresponding to a Hit@1 value of 100%. Note that the base rate probability for such a *Hit@1* for any given target word in our setup is extremely low, at $p = 1/N$. Since the individual responses themselves cannot be the target word, they were not considered for the rank computation.

The same procedure for computing neighborhood ranks and Hit@k values was applied to each dataset to ensure direct comparability of the results, with the only difference being the type of responses used to compute the centroids – free associations, feature generations, or word substitutions, depending on the dataset.

## Results

Following the evaluation setup described above, the Results section reports the computed Hit@k values over the systematically varied settings and (hyper)parameters:

- five datasets (four at the group level: taboo, Nelson, SWoW, Buchanan; plus one individual-level) with task settings ranging from single word substitutions to single and multiple free associations and multiple feature generation,
- two semantic models (fastText and word2Vec),
- two centroid calculation methods (type- or token-level, i.e., using each unique response once or as often as it was produced, respectively),
- four reference lexicon sizes (20,000, 50,000, 100,000, 250,000 words),
- response type (for the taboo dataset: only existing words, only novel words, or both).

For the group-level datasets, we also examine how neighborhood ranks vary with the number of unique and total responses per target, thereby addressing the question of how many responses are required to obtain an informative concept representation approximated by the response centroid. Presenting the frequency distributions of unique and total responses per target provides crucial context for interpreting the neighborhood ranks, as they reveal the variability in response distributions and clarify how representative the estimates are.

Since in the individual-level dataset (in contrast to the group-level dataset), there is a higher variability in the neighborhood ranks, we opt for presenting Hit@k values instead of neighborhood ranks to explore how many responses are needed to produce the best concept representations. Since in this dataset, responses were not averaged across participants, and each participant was required to produce ten unique responses per target by design, we only report the Hit@k distribution as a function of the total number of (unique) responses included in the centroid calculation (1–10). In addition, we present the distribution of neighborhood ranks across participants, along with a zoomed-in view of ranks (1–100), to examine how many participants produced responses whose centroids yielded lower ranks, indicating higher semantic proximity between the target and its centroid and, thus, more successful recovery of the intended concept meaning.

**Fig. 1:**
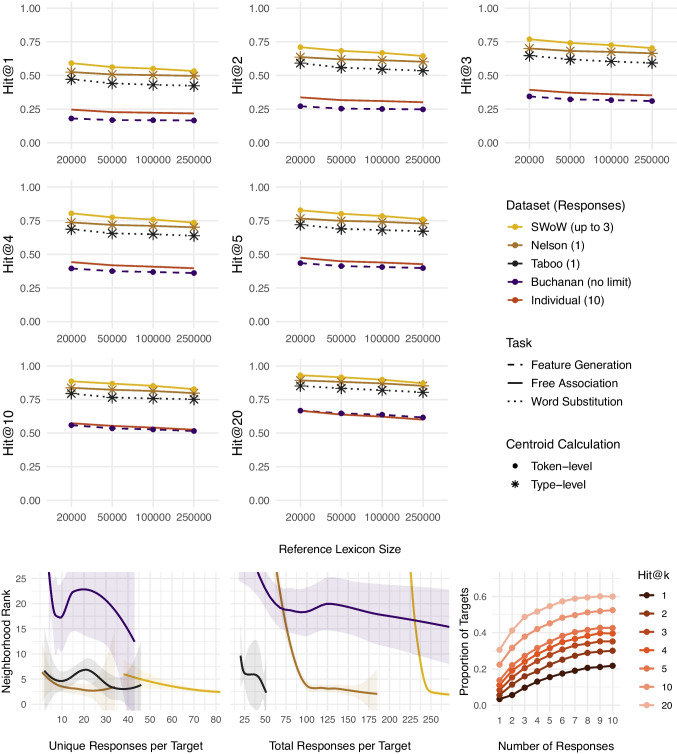
1) The fraction of targets that rank top-k (k = 1–20) in the neighborhood of their corresponding response centroid for the best-performing fastText models in each dataset. 2) Neighborhood ranks by number of unique and total responses per target for the best-performing fastText models for the group-level datasets at the 250k lexicon. 3) The fraction of targets that rank top-k (k = 1–20) as a function of the number of total (unique) responses per target for the best-performing fastText model for the individual-level dataset at the 250k lexicon

Before delving into the detailed analyses, we start with a high-level overview of the results for the best-performing fastText models across all five datasets and two centroid calculation methods. This enables a direct comparison of which parameter configurations perform best for each type of data in Fig. 1 and Table 1.

**Table 1:** Summary of centroid analysis outcomes for the best-performing fastText models across all five datasets and two centroid calculation methods at the 250k lexicon. The Hit@k values indicate the proportion of targets that rank top 1, 5, and 20 in the neighborhood of their response centroid. The responses/target needed indicate the number of unique and total responses per target that is needed to reach a neighborhood rank at which performance plateaus

Dataset	Centroid	Hit@1	Hit@5	Hit@20	Responses/Target Needed
SWoW	token-level	**53**%	**76**%	**87**%	Rank $\le$ 3 at 70 unique, 245 total
Nelson	type-level	50%	73%	85%	Rank $\le$ 3 at 17 unique,130 total
Taboo (existing)	type-level	42%	67%	80%	Rank $\le$ 3 at 35 unique, 48 total
Buchanan	token-level	18%	38%	57%	Rank $\le$ 18 at 37 unique, 160 total
Individual-level	token-level	22%	43%	60%	Plateaus at 8 total responses

### Overview: Best-performing models

Since across all task types—single-word substitutions (taboo), single and multiple free associations (Nelson and SWoW), and multiple feature generation (Buchanan)—fastText overall outperformed word2vec, the analyses presented in Fig. 1 and Table 1 employ the vectors obtained by using fastText for meaning-to-vector-mapping. The taboo results show only the word embeddings of the existing responses, as they performed better than the novel responses and, when combined, the novel and existing response types.

The first part of Fig. 1 presents the Hit@k values indicating how often the target concept appears among the top-k nearest neighbors (k = 1–20) of its corresponding response centroid. The results are shown across different reference lexicon sizes, defined by the number of most frequent English words (20–250k) whose cosine similarities with the target word were contrasted with the cosine similarity of the target and the response centroid to compute neighborhood ranks and subsequently Hit@k values.

Across all Hit@k thresholds and reference lexicon sizes, the highest Hit@k values are obtained for multiple free associations (SWoW), rendering this type of data most suitable for the successful recovery of the target meaning from the response centroid for the group-level data. Specifically, for the best-performing fastText model at the token level centroid calculation and the reference lexicon of 250k, we observe a Hit@1 of 53%, indicating that the target is the closest word to its response centroid in 53% cases (compared to the 250k most common English words), while Hit@20 rises to 87%. For the individual-level dataset with the same parameter and model setup, we observe a Hit@1 of 22% and Hit@20 of 60%.

Regarding the centroid calculation method, using each unique response at a type level (only once) shows better results for single-word substitution responses (taboo) and single-word free associations (Nelson). In contrast, for multiple free association responses (SWoW) and multiple feature generations (Buchanan), using each unique response at a token level (as often as it occurred) shows better results. This pattern indicates that the choice between weighting each unique response equally (type-level) or weighting more frequently produced responses more (token-level) in the centroid calculation should be determined by the structure of the data used in the analysis.

The second part of Fig. 1 examines the relationship between the neighborhood ranks and the number of unique and total responses per target used to compute the response centroids for the best-performing fastText models at the 250k-word lexicon for the four group-level datasets. This analysis addresses the question of the optimal number of responses required to achieve the most accurate estimation of the target concept representation. Importantly, a decrease in neighborhood ranks indicates an increase in model performance, with rank 1 corresponding to the target being the nearest semantic neighbor of its response centroid. We use local weighted regression (loess) with family=“symmetric” argument detecting and down-weighting the neighborhood rank outliers and *Z*-scores to detect and remove response number outliers at least 4 *SD* from the mean.

Our analysis indicates that, in general, the performance of all models improves as the number of unique and total responses increases. Note that the datasets differ substantially in the total and unique responses collected per target, making a direct comparison between them difficult. Nonetheless, in line with the Hit@k results, (multiple and single) free associations show lower and more stable rank distributions than the other types of group-level data examined. Specifically, to reach a plateau in performance with a neighborhood rank of $\le$ 3, the best-performing fastText model using token-level free associations needs around 70 unique and 245 total responses per target to map the target concept representations at the 250k reference lexicon.

The third part of Fig. 1 – the bottom-right panel – examines the individual-level data. It displays the relationship between the number of responses used to compute individual-level centroids and the proportion of cases in which the target word embedding appears among the top-k nearest neighbors (k = 1–20) of its corresponding response centroid. Here, higher values indicate better model performance, reflecting greater semantic proximity between the target and the response centroid and thus more accurate recovery of the intended concept meaning at the individual level. For the best-performing fastText model at the 250k lexicon, performance plateaus at approximately eight unique responses per target, suggesting that collecting more responses yields little additional benefit for the individual-level representations.

**Fig. 2:**
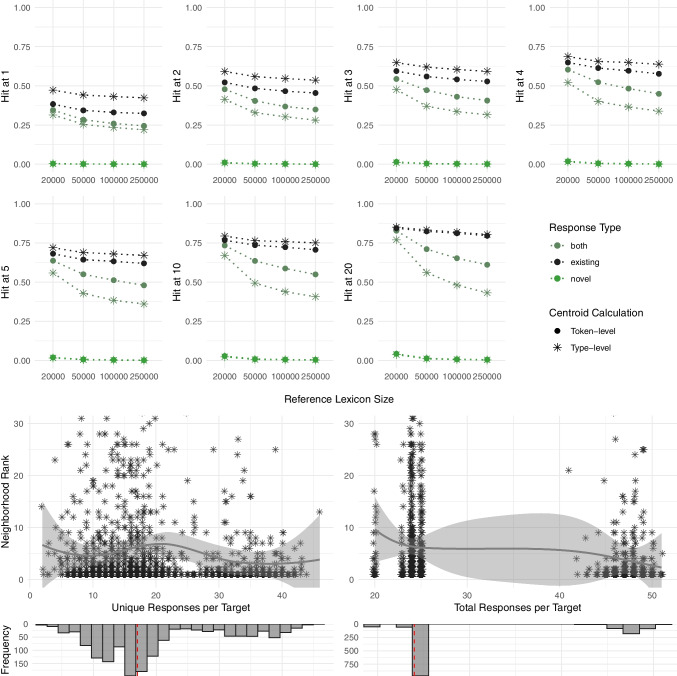
Taboo: 1) The fraction of the targets that rank top-k (k = 1–20) in the fastText neighborhood of their corresponding response centroid by response type, size of the reference lexicon, and centroid calculation method; 2) Neighborhood ranks by number of unique and total responses per target for the best-performing fastText model at the 250k lexicon (existing responses, type-level centroid calculation); 3) Distribution and median of unique and total responses per target

**Fig. 3:**
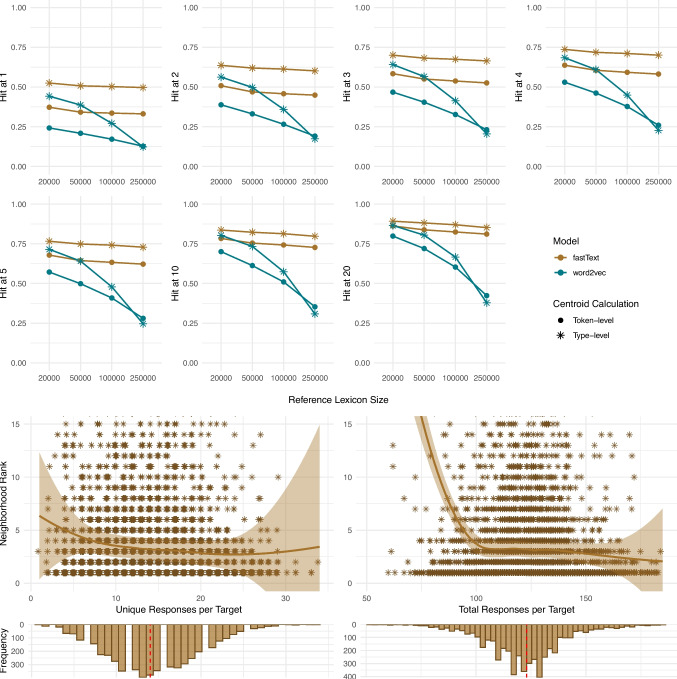
Nelson data: 1) The fraction of the targets that rank top-k (k = 1–20) in the neighborhood of their corresponding response centroid by model, size of the reference lexicon, and centroid calculation method; 2) Neighborhood ranks by number of unique and total responses per target for the best-performing model at 250k lexicon (fastText, type-level centroid calculation); 3) Distribution and median of unique and total responses per target

**Fig. 4:**
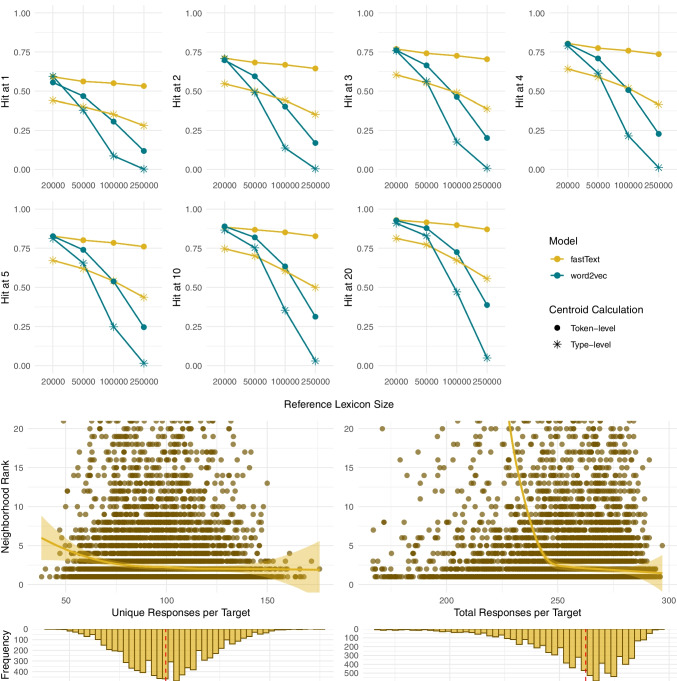
SWoW: 1) The fraction of the targets that rank top-k (k = 1–20) in the neighborhood of their corresponding response centroid by model, size of the lexicon, and centroid calculation method; 2) Neighborhood ranks by number of unique and total responses per target for the best-performing model at 250k lexicon (fastText, token-level centroid calculation); 3) Distribution and median of unique and total responses per target

**Fig. 5:**
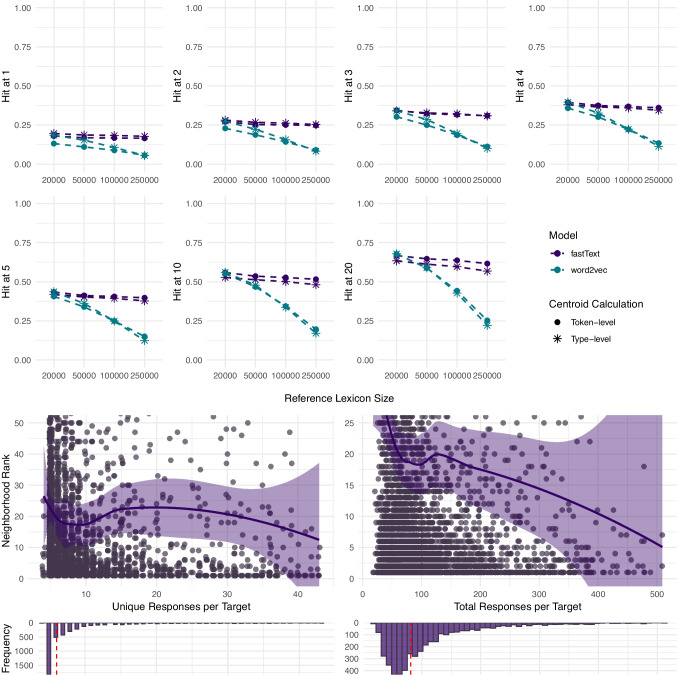
Buchanan data: 1) The fraction of targets that rank top-k (k = 1–20) in the neighborhood of their corresponding response centroid by model, size of the lexicon, and centroid calculation method; 2) Neighborhood ranks by number of unique and total number of responses per target for the best performing model at 250k lexicon (fastText, token-level centroid calculation); 3) Distribution and median of unique and total responses per target

**Fig. 6:**
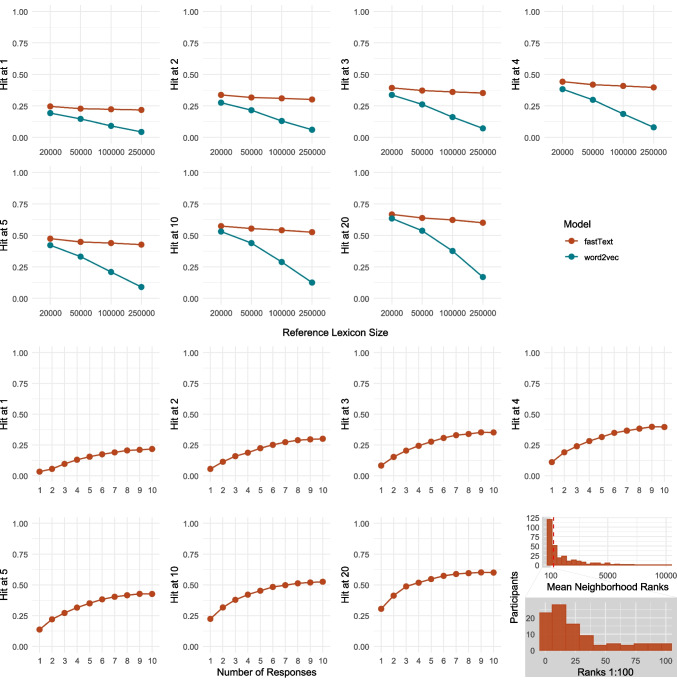
Individual-level centroids: 1) The fraction of targets that rank top-k (k = 1–20) in the neighborhood of their corresponding response centroid by model and size of the lexicon; 2) Hit@k values by number of responses used in the centroid calculation for fastText as the best performing model at 250k lexicon; 3) Mean and median neighborhood rank by number of participants for fastText at 250k lexicon on scales 1:10,000 and 1:100

Figures 2, 3, 4, 5 and 6 in the next four subsections plot the results for each dataset separately, allowing for the comparison of the fastText and word2vec DSMs in the first part and inviting a closer look at the neighborhood rank distribution across different numbers of unique and total responses per target in the second part. The frequency distribution of unique and total responses per target in the third part examines the underlying variation in response numbers and allows a more informed interpretation of the second part. Figure 2, plotting the results for the taboo dataset, further distinguishes between novel responses, existing responses, and their combination. In contrast to the other datasets, Fig. 6, which presents the individual-level data, plots the Hit@k distribution over the number of responses that were used in the centroid calculation and neighborhood rank distribution and the number of participants whose response centroids yielded low ranks (1–100), indicating high proximity between the target and its corresponding centroid in semantic space.

### Analyses of individual datasets

#### Group-level datasets

##### Taboo dataset

Figure 2 uses Hit@k values determined via neighborhood ranks to demonstrate to what extent the intended meaning of a concept can be recovered from the average of a set of responses when the original concept label cannot be used to refer to the concept by design. The results are presented for the best-performing fastText model only, as its use of subword information enables the representation of novel words as vectors, in contrast to word2vec, which does not support this type of analysis. For both centroid calculation methods and across all lexicon sizes, the centroids of existing word responses consistently rank higher in the neighborhood of their corresponding centroids (compared to the centroids of novel word responses and both novel and existing response types taken together). In line with Pugacheva and Günther (2024), this implies that in a word substitution involving single-word responses, existing word responses are better suited for locating the intended concept meaning in a multidimensional semantic space (see also Gatti et al. (2024), who demonstrated that novel and existing words occupy different sections of the semantic space). For existing word responses, the model performance is higher when each unique response is used once in the centroid calculation (type-level) compared to when responses are considered as often as they occur in the data (token-level). However, the magnitude of this improvement diminishes slightly at greater Hit@k as the model’s overall performance increases. This trend is reversed for the novel word responses (minimally) and both types of responses combined. To conclude, for the best-performing setup at the reference lexicon of 20,000 – fastText with existing words and each response used once in the centroid calculation – we observe a Hit@1 of 47%, Hit@5 of 72%, and Hit@20 of 85%. With the same model setup, at the reference lexicon of 250,000, we observe a Hit@1 of 42%, Hit@5 of 67%, and Hit@20 of 80%.

The second part of Fig. 2 illustrates the relationship between the neighborhood ranks and the number of unique and total responses per target used in the centroid calculation performed at the type level as this hyperparameter has shown the best results for the reference lexicon size of 250,000 words. As evidenced, the performance of the model increases with the increasing number of unique and total responses (note that a *decrease* in neighborhood ranks indicates an *increase* in model performance), reaching the neighborhood rank of 5 at 10 unique and 44 total responses per target. It reaches the neighborhood rank of 3 at 35 unique and 48 total responses per target, providing a practical guideline for the number of responses to collect. The third part of Fig. 2 shows that with a range from 2 to 46^4^, 25% and 50% of the unique responses per target fall below 13, 50% – below 17, and 75%– below 23. The total responses per target values range from 20 to 51, with 25% of the data below the median of 24 and 75% below 44. This suggests that the proposed response thresholds are based on sufficient data.

##### Nelson dataset

Figure 3 demonstrates the robustness of the centroid analysis as a method to estimate the underlying concept representation from a set of single-word free association responses. For the Nelson association data, the choice of model significantly affects the target word’s rank within the centroid neighborhood, with fastText consistently outperforming word2vec across all evaluation metrics. The performance gap between the two models widens considerably as the neighborhood size increases at both the type- and the token-level centroid calculation methods. The performance of both fastText and word2vec models is generally higher when each unique response is used once in the centroid calculation (at the type level) than when responses are considered as often as they occur in the participant data. For fastText, this pattern persists across all Hit@k considered, although the magnitude of improvement diminishes slightly at higher Hit@k as the model’s overall performance increases. For word2vec, the advantage of using unique responses only once in the centroid calculation is more pronounced for smaller lexicon sizes. It reaches a marginally reverse pattern at a lexicon of 250,000 words across all Hit@k. As the reference lexicon size increases, both models show a decline in performance; however, fastText shows a substantially smaller decline, maintaining higher scores across all Hit@k compared to word2vec. Ultimately, at a lexicon size of 20,000, for fastText and the type-level centroid calculation method as the best-performing model, we observe a Hit@1 of 53%, Hit@5 of 77%, and Hit@20 of 89%. For the lexicon size of 250,000 and the same model setup, we observe a Hit@1 of 50%, Hit@5 of 73%, and Hit@20 of 85%.

The second part of Fig. 3 demonstrates that at the lexicon size of 250,000 words, the efficiency of the best-performing model for the Nelson single-response association data (fastText, type-level centroid calculation) starts to plateau, reaching the neighborhood rank value of 3 at around 17 unique responses and 130 total responses per target, indicating that this range provides a reasonable benchmark for data collection. As shown in the third part of Fig. 3, with the range of 1 to 34 and a median of 14, 25% of unique responses per target fall under 11 and 75% of data fall under 18. Total responses per target range from 52^5^ to 185, with 25% of data below 112, a median of 123, and 75% of data below 132. This suggests that our estimates of the required number of responses are based on a sufficient number of observations.

##### SWoW dataset

Consistent with the Nelson single-response data, the SWoW data containing up to three association responses per target word demonstrates that fastText tends to outperform word2vec across all metrics and ranks, particularly when the reference lexicon size is large (see Fig. 4). For fastText, this improvement persists across all ranks, with its magnitude growing slightly at higher ranks. Word2vec shows a similar pattern with a slightly reverse effect at lower Hit@k and smaller lexicons. For instance, at the lexicon size of 20,000, word2vec with the type-level centroid calculation method shows comparable results to fastText with the token-level centroid calculation method, producing Hit@1 of 59% (vs. 59% for fastText), Hit@5 of 81% (vs. 83% for fastText), and Hit@20 of 91% (vs. 93% for fastText). Naturally, as the lexicon size increases, both models show a performance decline, but the decline is considerably more pronounced for word2vec. For fastText, as by far the best-performing model at the 250,000-word lexicon and the token-level centroid calculation method, we observe a Hit@1 of 53%, Hit@5 of 76%, and Hit@20 of 87%. In contrast to the Nelson data, both models generally perform better when unique responses are used as often as they are produced in the centroid calculation. Given that the difference between the Nelson and SWoW datasets lies in the number of responses collected per participant, it is reasonable to infer that this factor drives the performance difference between type- and token-level centroid calculation methods. Note that centroid analysis shows overall better absolute performance when applied to SWoW dataset.^6^

As to the number of responses, as the best-performing model at the 250,000-word lexicon, fastText with token-level centroid calculation method, starts to plateau reaching the neighborhood rank value of 3 at around 70 unique and 245 total responses per target providing a practical target for achieving a reliable model performance. As shown in the third part of Fig. 4, with the range of 38 to 175, 25% of unique responses per target fall under 84, 50% under 98 and 75% under 112. Total responses per target range from 165^7^ to 297 with 25% of data below 246, a median of 262, and 75% of data below 273. This implies that the number of responses is supported by a sufficient amount of data.

##### Buchanan dataset

As shown in Fig. 5, for the Buchanan feature norms data (where participants were asked to produce as many responses per target as possible), using fastText to extract the word vectors generally results in better neighborhood ranks compared to word2vec. Consistent with previous results, word2vec performs worse overall, especially for the larger lexicon sizes. For both models, both centroid calculation methods (i.e., type- and token-level) yield comparable results. Overall, considering responses at a token level in the centroid calculation results in slightly better performance with a minor reverse pattern at lower Hit@k for fastText and a minor reverse pattern at higher lexicon sizes for word2vec. For instance, at a 20,000-word lexicon fastText, the type-level centroid calculation produces the best results at lower Hit@k- a Hit@1 of 20% and Hit@2 of 28%-, whereas word2vec, the type-level centroid calculation produces the best results starting with Hit@4- a Hit@4 of 40%, Hit@5 of 44%, and Hit@20 of 68%. At the lexicon size of 250,000, the overall best-performing model, fastText with the type-level centroid calculation, produces a Hit@1 of 18%, Hit@5 of 38%, and Hit@20 of 57%. It reaches the neighborhood rank value of 18 the first time at around 10 unique and 100 total responses per target, and after a slight increase, the second time at around 37 unique and 160 total responses per target. As shown in the third part of Fig. 4, with the range of 4 to 43^8^, 25% of unique responses per target fall under 5, 50% under 6 and 75% under 9. Total responses per target range from 18 to 509 with 25% of data below 56, a median of 81, and 75% of data below 128. This suggests that the range of 37 unique and 160 total responses per target is a more reliable recommendation as it relies on enough data, and including additional responses contributes little to improving the neighborhood ranks.

### Individual-level dataset

The upper part of Fig. 6 illustrates the rates of the target words being the closest word to their corresponding centroids calculated by averaging the free associations per item per participant, with ten unique responses collected for each item. The lower part of Fig. 6 presents the Hit@k values when only taking into account the first 1, 2, 3,..., or all ten responses to examine if the increase in the number of responses influences the rank values. The lower-rightmost graph displays the number of participants whose responses yielded neighborhood ranks ranging from 1 to over 10,000, and a zoom-in on the rank scale from 1 to 100.

Consistently with the group-level results, fastText performs better compared to word2vec, resulting in better neighborhood ranks across all reference lexicon sizes, with a minor decline in performance for fastText and a major decline in performance for word2vec observed at larger lexicons. Specifically, for fastText at a lexicon size of 20,000, we observe a Hit@1 of 25%, Hit@5 of 47%, and Hit@20 of 67%. For the lexicon size of 250,000 and the same model setup, we observe a Hit@1 of 22%, Hit@5 of 43%, and Hit@20 of 60%.

The lower graphs in Fig. 6 for fastText as the best performing model show that, at the lexicon size of 250,000 words, the increase in the number of responses used in the centroid calculation positively correlates with the Hit@k values. This suggests that an increase in the number of responses positively contributes to the precision of the model in estimating the underlying concept representations, but in a decelerated manner with a plateau at eight responses, indicating that this number provides sufficient information. The graph illustrating the mean neighborhood rank distribution reveals notable variation among participants in how high the target words rank in the neighborhood of the response centroids indication that some response sets are more informative than others. With a mean neighborhood rank per participant ranging from 1.2 to 11,286.7^9^, 25% of the data are below 30.9, 50% below 327.3 and 75% below 1385.3.

## Discussion

The present research examines response centroid calculation as a method to retrace concepts, represented as vectors in a multidimensional semantic space, from open-ended participant responses. At the group level, concept representations are derived from responses across participants and items. This approach can be used to compare conceptual organization across different populations and examine how factors such as language, culture, cognitive differences, or experimental manipulations influence shared representations. On an individual level, this analysis measures response centroids per item per participant and, thus, provides a quantitative framework for assessing variability in conceptual representations between individuals or even within the same individual across different contexts or experimental conditions. This allows for fine-grained assessments of how personal experiences, expertise, cognitive styles, or even temporary contextual influences affect conceptual representations.

### Summary of results and recommendations

As mentioned in the section *Best performing models* in the *Results* and demonstrated in Figs. 2, 3, 4, 5 and 6, on the dataset level, centroid analysis as a method to retrace the meaning of a concept from participant responses demonstrates overall better results when meaning-to-vector mapping for the responses and target words is performed using the fastText model (compared to word2vec). This is the case for responses in the form of single-word substitutions (taboo dataset), single and multiple free associations (Nelson and SWoW datasets), and multiple feature generation (Buchanan dataset), especially at larger reference lexicon sizes. As the reference lexicon size increases from 20,000 to 250,000, naturally and inevitably, both models in all the datasets show a decline in Hit@k, but this decline is considerably more pronounced for word2vec. In the present research, we systematically tested different reference lexicon sizes to carefully examine the robustness of our results. Realistically, however, native speakers of American English aged 20 to over 60, from both graduate and non-graduate populations, are estimated to have receptive vocabularies ranging from 27,100 to 46,400 lemmas known and productive vocabularies comprising less than half of their receptive word knowledge (Brysbaert et al., 2016). Thus, the reference lexicon sizes between 20,000 and 100,000 (considering that different speakers in a group know different words) likely best align with what participants can actually be expected to know and produce.

Regarding the centroid calculation method, the optimal approach depends on task setting, with type-level centroids showing better results for single-response tasks and token-level centroids for multiple-response tasks (see Fig. 1 and Table  1). In addition to the strong connections captured by the single-word responses, collecting multiple responses for a target word allows capturing weaker connections between words in a semantic network (De Deyne et al., 2013). The latter result thus suggests that for a dataset containing a number of weak associates due to the task setting, adding the weight to participant responses by considering them in the centroid calculation as many times as they were produced improves the effectiveness of the centroid analysis method. Following this logic, for datasets containing mostly strong associates by design, considering each unique response only once (at a type level) and thus particularly emphasizing the information provided by rare responses, yields better results.

Similarly, the task setting and the centroid calculation method need to be considered when deciding on the number of unique and total responses to be collected for a given purpose. Figures 1, 2, 3, 4, 5 and 6 indicate that, in general, the performance of all models improves as the number of unique and total responses increases – albeit in a decelerated manner, with diminishing returns after a certain number of responses is reached. However, there is variation between the datasets (Fig. 1) that can be at least partially caused by differences in the number of unique and total responses available. For single-word substitutions (taboo data) and the type-level centroid calculation method, the best-performing model reaches the neighborhood rank of 3 at approximately 35 unique (ranging between 2 and 46) and 48 (ranging between 20 and 51) total responses per target. For single-response associations (Nelson data) in the same set-up, the model reaches rank 3 at 17 unique (ranging between 1 and 34) and 130 (ranging between 52 and 185) total responses. For the multiple free associations (SWoW data) and the token-level centroid calculation method, the model achieves the neighborhood rank mean of 3, at a higher number of approximately 70 unique (ranging between 38 and 175) and 245 total (ranging between 165 and 297) responses per target need to be collected. In contrast, for multiple feature generations (Buchanan data) and the token-level centroid calculation method, approximately 37 unique (ranging between 4 and 43) and 160 total (ranging between 18 and 509) responses per target still result in a much lower neighborhood rank of 18.

To summarize, for the reference lexicon size of 250,000 words, the best method to recover the response-generating concept as a vector in a multidimensional semantic space from the averaged vectors of participant responses *at the group level* is to:

- collect multiple free associations (e.g., 3 associations as in the SWoW dataset),
- collect at least 245 total responses per target (aiming for 70 unique responses from participants),
- use the fastText model to extract the word vectors for responses and the targets, and to
- consider each response in the centroid calculation as often as it occurred in the data (at the token level).

For the centroid analysis at the individual level, the best method according to our results is to:

- collect eight responses per participant per target, at which point performance plateaus and to
- use the fastText model to extract the word vectors for responses and the targets.

Note that, by instruction, each unique response should only be produced once per participant.

Compared to the best-performing model at the group level, the individual-level centroid analysis appears to be less precise for measuring the concept meaning.

However, here we also need to assume that our evaluation gold standard – the vector representation of the target word – is less adequate than for the group-level analysis: After all, this distributional vector is by itself an averaged group-level representation, which will be less accurate in capturing the varying true semantic representations of individual speakers than the average representation of entire speaker groups. It thus appears likely that the results presented here underestimate the method’s accuracy for individual-level representations that would be more accurately measured using the individual-level golden standard target words whose vector representations are to be recovered from the individual-level responses.

### Application of the method

Centroid analysis is especially useful as an economical method to map a small number of concepts/semantic representations into a pre-existing semantic space, such as a distributional vector space. To highlight this, we compare the method to other approaches for mapping semantic representations.

Another prominent approach to map semantic representations is to infer the underlying representations from behavioral data, such as free associations or similarity judgments (Aeschbach et al., 2025; De Deyne et al., 2017; Hebart et al., 2020; Kennett et al., 2017; Roads & Love, 2021). While this provides high-quality measurements of semantic memory (often in the form of semantic networks), it requires a relatively large number of responses, and only allows for comparisons between items for which data was actually collected. This is especially daunting when one wishes to map semantic representations of individual speakers, where this large amount of data has to be collected for each individual (Aeschbach et al., 2024). In comparison, the centroid analysis method can be applied for individual items, even within individual speakers, which can then be readily compared to all other entries in the pre-existing semantic space.

The method then allows for a variety of quantitative comparisons that can be highly useful for different research questions; for instance, we can compare the representations:

- for the same concept between different groups (e.g., left-leaning or right-leaning voters; younger and older individuals) or even between different individuals,
- for the same concept between experimental conditions (e.g., presented with a positive versus negative framing),
- between different concepts within the same groups or even within the same individual,
- between a concept and *all other concepts* already represented in the pre-existing semantic space.

In the present evaluation setup, we validated centroid analysis by recovering semantic representations for lexicalized concepts (i.e., existing word meanings). This was done because an objective gold standard is available in this scenario – the respective word embeddings of these lexicalized concepts. However, the most promising and relevant intended use case for centroid analysis lies beyond this setting, in more open scenarios. For example, one may ask participants to produce the first ten words that come to their mind when thinking about their life as a whole or about their view of the future, in order to estimate a representation for those idiosyncratic concepts. Alternatively, one may collect associations for images of novel concepts or ambiguous images, for literary texts, for pop songs, or for pieces of artwork from groups of participants to estimate their respective concept representations.

Beyond serving as a measurement tool, centroid analysis can also be applied as an analysis tool for open responses. For example, in the domain of free association data, some previous studies have stated that word embeddings capture free association data relatively poorly (De Deyne et al., 2017; De Deyne et al., 2016; Kennett et al., 2017; see however Nematzadeh et al., 2017; Thawani et al., 2019). However, when moving beyond the similarities between the target and individual-produced (or non-produced) associations, our present results, especially on the SWoW data (De Deyne et al., 2019) show that all responses taken together, in form of the response centroids, are very often highly diagnostic of the target. As described in the introduction, this is in line with a spreading activation account of language production, where the source of the spreading activation can be recovered from the items it activates. However, we still have to acknowledge that this analysis only focuses on the responses that *were* actually produced and cannot account for why many close neighbors of the target are *not* produced. We suspect that other factors besides semantic similarity, such as frequency effects, are at play here.

## Data Availability

All experimental materials are available at https://osf.io/tq6ja/

## Footnotes

1 Hit@k quantifies how often the target appears in the top k neighborhood ranks

2 And one outlier with 63 unique and 105 total responses.

3 We used the strength.SWOW-EN.R123.csv file of the SWOW-EN18 dataset, available at https://smallworldofwords.org/de/project/research#download.

4 The outlier with 63 unique and 105 total responses per target was manually excluded from the response number analysis.

5 Four outlier items at least 4 *SD* from mean, ranging between 15 and 47 total and 1 and 34 unique responses were excluded from the response number analysis.

6 It’s worth mentioning again that SWoW contains 2,075,318 responses to 8091 different target words whereas Nelson dataset contains 602,832 responses to 4080 different target words.

7 36 outlier items at least 4 *SD* from mean that received between 56 and 162 total and 32 to 101 unique responses were excluded from the response number analysis.

8 63 outliers whose distinct or total response values were at least 4 *SD* from the mean (with values ranging between 10 and 62 unique and 107 and 1951 total responses) were excluded from the response number analysis.

9 Two neighborhood rank outlier items at least 4 *SD* from mean, 13,491.7 and 34,719.1, were excluded from the response number analysis.

## Code availability

All analysis scripts are available at https://osf.io/tq6ja/. To make centroid analyses easily available to other researchers, we included the centroid_analysis() function in the latest version of the *LSAfun* package for R (Günther et al., 2015). This function takes as input a set of responses and a vector space, and allows users to specify whether all or only unique responses should be considered. If no target words are provided, this function simply calculates the centroid of the responses. If target words are provided, the function also computes – for each target word – the cosine between target and centroid, the rank of the target in the neighborhood of the centroid, and the rank of the centroid in the neighborhood of the target. To calculate ranks, a reference list of the other words for which ranks should be computed can be provided.

## Declarations

### Open Practices Statement

Most of the data analyzed in this study (except for the individual-level data) was sourced and adapted from existing studies in the literature. Neither of the analyses reported was formally preregistered.
